# Decreased interferon regulatory factor 6 expression due to DNA hypermethylation predicts an unfavorable prognosis in clear cell renal cell carcinoma

**DOI:** 10.7150/jca.62394

**Published:** 2021-09-13

**Authors:** Zhi Li, Wuping Yang, Jianhui Qiu, Haozhe Xu, Bo Fan, Ke Li, Jingcheng Zhou, Yuan Li

**Affiliations:** 1 Department of Urology, Xiangya Hospital, Central South University, Changsha, 410008, Hunan, China.; 2 Department of Urology, Peking University First Hospital, Beijing 100034, P.R. China.; 3 Department of Urology, the Second Xiangya Hospital, Central South University, Changsha, 410011, P.R. China.

**Keywords:** ccRCC, IRF6, DNA hypermethylation, prognosis, immune cells infiltration

## Abstract

**Background:** Emerging evidences have indicated that IRF6, as a member of the Interferon regulatory factors (IRFs) family, plays important roles in a variety of tumors. However, the expression status of IRF6 and its prognostic value in clear cell renal cell carcinoma (ccRCC) remain unclear.

**Methods:** In this study, we used TCGA-KIRC, GEO and TIP databases and immunohistochemistry staining to determine the expression profile, clinico-pathological features and prognostic value of IRF6 in ccRCC. MSP and demethylation analysis were utilized to verify the regulatory effect of DNA methylation on IRF6 expression.

**Results:** Our results found that IRF6 expression was downregulated in ccRCC tissues and cell lines, and decreased IRF6 expression was associated with worse clinicopathological features and poorer prognosis. Besides, the results of multivariate Cox regression analysis also confirmed that decreased IRF6 expression was an independently risk factor predictor of shorter Overall Survival (OS) (HR: 0.8524, 95%CI: 0.7614-0.9543, P=0.0056) and Disease Free Survival (DFS) (HR: 0.7024, 95%CI: 0.6087-0.8104, P<0.0001) in ccRCC patients. Moreover, the results of MSP and demethylation analysis validated that decreased IRF6 expression was caused by DNA hypermethylation. Furthermore, our results showed that IRF6 expression was associated with the infiltration levels of multiple immune cells in ccRCC.

**Conclusions:** These findings demonstrated that IRF6 expression was significantly reduced in ccRCC and DNA hypermethylation played an important role in decreased IRF6 expression. In addition, the decrease of IRF6 was related to the unfavorable prognosis of ccRCC patients and the alterations of tumor immune cells infiltration.

## Introduction

Kidney cancer is one of the three major tumors of the genitourinary system, affecting 431,288 new individuals and causing 179,368 new deaths in 185 countries worldwide in 2020 1. Renal cell carcinoma (RCC) is the most primary type of kidney cancer, accounting for up to 85% of the cases. Clear cell RCC (ccRCC) is the most common RCC subtype, occurring in 70% to 75% of the cases, and is closely related to von Hippel-Lindau (VHL) gene alterations 2. In the past decade, the medical treatment of RCC has transitioned from cytokine approach to targeted therapy, and now it has transitioned to new immunotherapy agents 3, 4. Although the 5-year relative survival rates at diagnosis have improved to some extent, the state quo of overall prognosis of ccRCC patients remains poor, especially for patients with advanced tumors 4, 5. Therefore, it is urgently to find more effective and safer therapeutic targets to improve the survival outcome of ccRCC patients.

Interferon regulatory factors (IRFs), known as a family of transcriptional regulators, play vital roles in several processes, such as inflammation, cell differentiation and development, regulation of host defense against pathogens and tumorigenesis 6-11. The family comprises nine members (IRF1-9) and typically recognize the promoter composed of the IRF consensus sequence 5'-GAAA-3' 12. Recent studies have shown that IRF6, as a member of the IRFs family, plays an important role in the occurrence and development of a variety of tumors. For example, IRF6 predicts a favorable prognosis in gastric cancer 13; IRF6 distinctively reverses stemness phenotype in nasopharyngeal carcinoma 14; IRF6 downregulation promotes squamous cell carcinoma (SCC) cell invasive and reintroduction of IRF6 into SCC cells inhibits cell growth 15; miR-587 promotes cervical cancer by repressing IRF6 16. However, the expression pattern and clinicopathological role of IRF6 and its prognostic value in ccRCC remain unclear.

In this study, we presented the expression profile and the prognostic role of IRF6 in ccRCC and its relationship with clinicopathological features and infiltration of immune cells using the data from TCGA-KIRC, GEO DataSets and TIP databases. In addition, the expression of IRF6 protein was further confirmed in 50 ccRCC tissues and 20 matched adjacent normal renal tissues using immunohistochemistry staining, which validated the prognostic value and clinicopathological roles of IRF6 in ccRCC. Furthermore, we verified that DNA hypermethylation played an important role in decreased IRF6 expression in ccRCC.

## Materials and methods

### Bioinformatic data mining

RNA-sequencing and clinicopathological data of 5 Gene Expression Omnibus (GEO) datasets (GSE40435, GSE53757, GSE66272, GSE126964, GSE73731) were obtained from GEO DataSets (https://www.ncbi.nlm.nih.gov/gds). RNA-sequencing, DNA methylation, clinicopathological and survival data of TCGA-KIRC (The Cancer Genome Atlas-Kidney Renal Clear Cell Carcinoma) were also downloaded (https://portal.gdc.cancer.gov/). The infiltration data of 14 kinds of immune cells in TCGA-KIRC was obtained from TIP -- Tracking Tumor Immunophenotype (http://biocc.hrbmu.edu.cn/TIP/index.jsp).

### Immunohistochemistry staining

This study was approved by the Biomedical Research Ethics Committee of Xiangya Hospital, Central South University, and written informed consents were also obtained from all patients. A total of 70 paraffin sections, including 50 ccRCC tissues and 20 matched adjacent noncancerous tissues were obtained from patients diagnosed with ccRCC at Xiangya Hospital from 2014 to 2016. In order to extremely eliminate effect of some other confounding factors, patients who only received puncture surgery, or diagnosed with malignant tumors of other organs or systems, or combined with severe underlying diseases, such as cardiac dysfunction (> grade 2), respiratory dysfunction PaO2 < 70 mmhg or (and) CO2 > 45mmhg, liver dysfunction: Child-Pugh is C, were excluded in our study. Finally, the 70 paraffin sections, including 50 ccRCC tissues and 20 matched adjacent noncancerous tissues were obtained from the Department of Pathology at Xiangya Hospital and used for immunohistochemical staining to detect the protein expression level of IRF6, and the prognostic information of these patients was also obtained through follow-up. The specific primary antibody information was as follows: anti-IRF6 (1:100, AF2557, Beyotime Biotechnology, China).

Image acquisition and analysis: after immunoperoxidase labeling, IRF6 expression intensity was assessed by estimating the area of the objects and the medium pixel intensity per object, as the integrated optical density (IOD). To analyze the IOD of IRF6, five visual fields (per immunohistochemical section) were randomly selected under high magnification (10 × 40) and photographed. All images were acquired and processed in TIFF format, analysis was done using the Image_Pro_Plus analysis system with high-resolution and multicolor imaging. The same light level as for incidental light without a slide was kept for each image acquired. Then, taking the average value of IOD as the cut-off value, the 50 ccRCC patients were divided into two groups: low expression group and high expression group. Finally, in order to determine the relationship between the expression level of IRF6 and the survival outcome of ccRCC patients, K-M survival curve was made according to the results of follow-up.

### Western blot

The protein of ccRCC cell lines (786-O, OSRC2, Caki-1 and A498) and the normal human renal tubular epithelial cell line HK2 was extracted using RIPA buffer, and the total protein levels were quantified by BCA method. Protein (30 μg per lane) was separated by SDS-PAGE and transblotted to PVDF membranes. Then the membranes were blocked in 5% nonfat milk powder and incubated overnight at 4 °C with anti-IRF6 (1:1000, AF2557, Beyotime Biotechnology, China). All membranes were stripped and incubated with anti-GAPDH antibody (1:8000, Proteintech, China) again as a loading control.

### Methylation-specific PCR (MSP) and demethylation analysis

MethPrimer 2.0 was used to predict the CpG islands of IRF6 DNA and design the corresponding primers. Genomic DNA was extracted from cell lines, the purified DNA was exposed to bisulfite using a DNA Bisulfte Conversion Kit. Amplified PCR products were separated by 2% agarose gel electrophoresis and visualized with GelRed. The specific primer sequences used for MSP were as follow: methylated primer sequences (5'-3'): forward: GTGGTTATATTTGGGAGGCG, reverse: AACTACAAATTCCTCTCCCCG; unmethylated primer sequences (5'-3'): forward: GTGGTTATATTTGGGAGGTG, reverse: AAACTACAAATTCCTCTCCCCAT.

OSRC2 and Caki-1 cells were seeded in six-well plates and treated with 5 μM 5-Aza-2′-deoxycytidine (5-Aza-dC, A, Sigma-Aldrich) for 4 days. Besides, cells were cultured with or without 100 Nm Trichostatin A (TSA, T, Sigma-Aldrich) for the final 24 h. Then DNA was isolated for IRF6 MSP and protein was extracted for western blot.

### Statistical analysis

Differences in continuous variables were tested by Non-parametric Mann-Whitney. Kaplan-Meier curve and log-rank test were applied to examine the prognostic value of IRF6 in ccRCC. The univariate and multivariate Cox regression analyses of IRF6 were also performed. The correlation between IRF6 expression and the DNA methylation levels of its CpG sites was assessed by Pearson's correlation test. The correlation between IRF6 expression and the infiltration levels of immune cell and the expression of immune cell markers was also examined. A P-value < 0.05 indicated statistical significance. All data were analyzed using Graphpad prism 7.0 and R language.

## Results

### IRF6 expression was decreased in ccRCC based on GEO and TCGA-KIRC data

**Figure 1:**
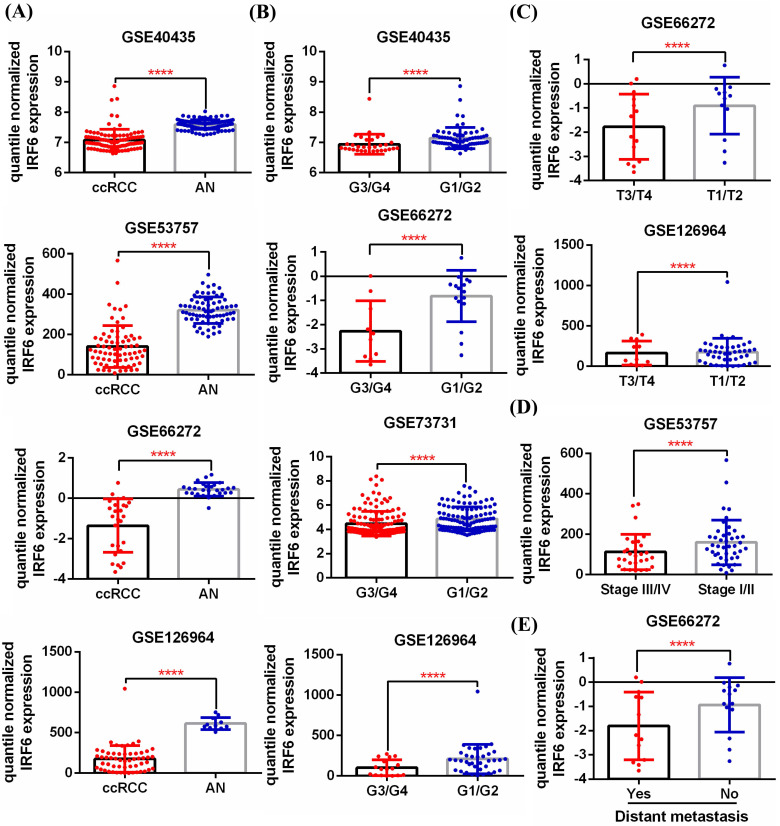
** IRF6 expression was downregulated in ccRCC based on GEO datasets. (A)** IRF6 expression was significantly decreased in ccRCC than that in adjacent normal renal tissue. **(B-E)** Decreased IRF6 expression was associated with higher histological grade, advanced tumor stage, higher pathological stage and distant metastasis. ****p < 0.0001.

**Figure 2:**
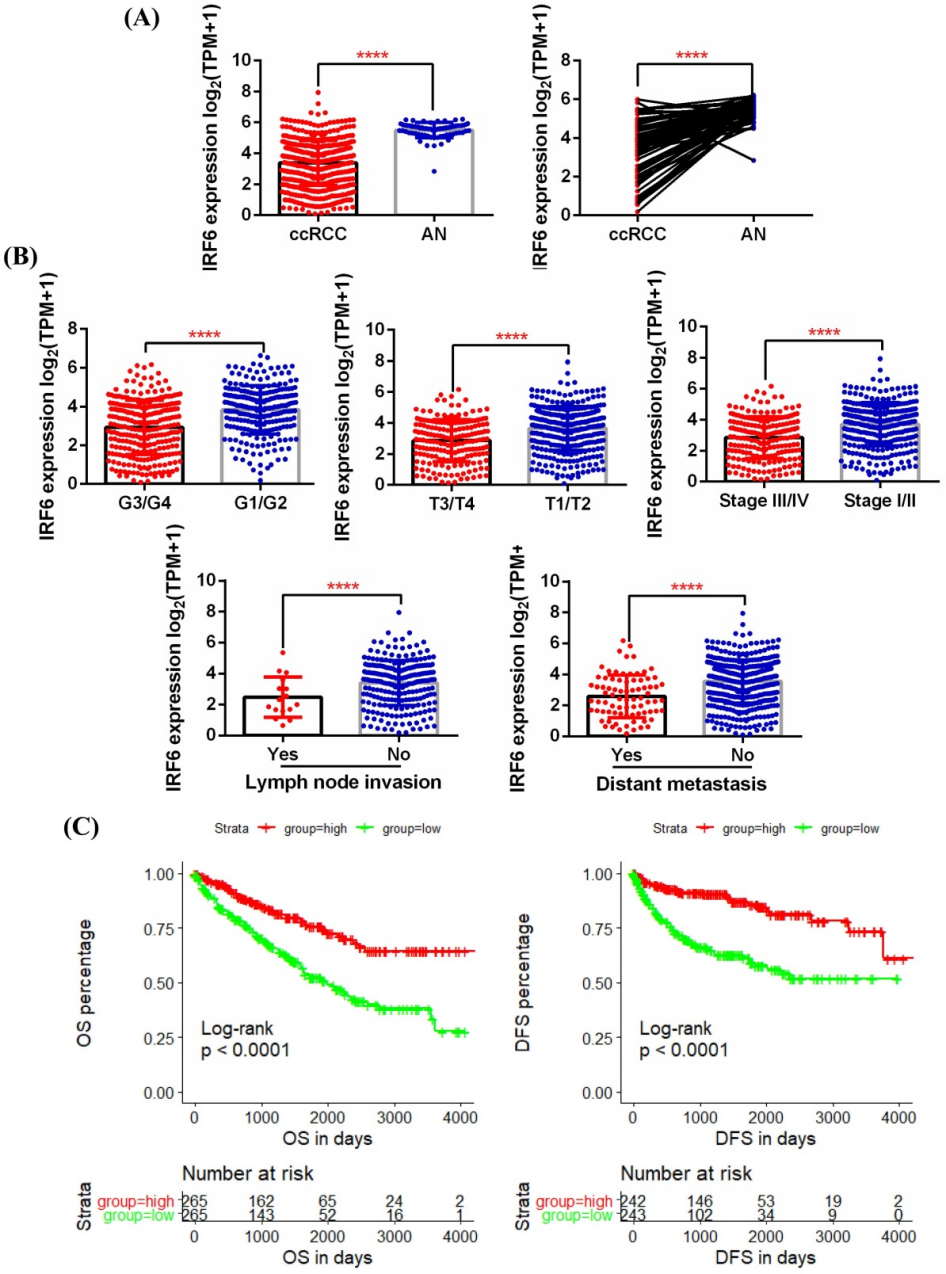
** Decreased IRF6 expression was associated with worse pathological features and poor prognosis based on TCGA-KIRC data. (A)** IRF6 expression was significantly decreased in ccRCC. **(B)** Decreased IRF6 expression was associated with higher histological grade, advanced tumor stage, higher pathological stage, lymph node invasion and distant metastasis. **(C)** IRF6 low expression group have shorter OS and DFS compared to IRF6 high expression group. Patients were separated into two groups according to the median cutoff of IRF6 expression. ****p < 0.0001.

First, 5 GEO datasets (GSE40435, GSE53757, GSE66272, GSE126964, GSE73731) were used to analyze the expression pattern of IRF6 in ccRCC and its correlation with different pathological features. Results showed that IRF6 expression, compared with adjacent normal renal tissues, was significantly decreased in ccRCC (Figure 1A), and decreased IRF6 expression was related to higher histological grade (G3/G4) (Figure 1B), advanced tumor stage (T3/T4) (Figure 1C), higher pathological stage (Stage III/IV) (Figure 1D) and distant metastasis (Figure 1E). Besides, the RNA-seq data of 539 ccRCC tissues and 72 adjacent normal tissues from TCGA-KIRC was utilized to validate the expression status of IRF6 in ccRCC. Consistent with the analysis results of GEO data, IRF6 expression was remarkably reduced in ccRCC compared to adjacent normal renal tissues based on TCGA-KIRC data (Figure 2A). Moreover, reduced IRF6 expression was also associated with worse pathological characteristics, including G3/G4, T3/T4, Stage III/IV, lymph node invasion and distant metastasis (Figure 2B).

### Decreased IRF6 expression independently predicted shorter OS and DFS in ccRCC patients based on TCGA-KIRC data

In order to test the effect of IRF6 expression on the prognosis of ccRCC, we obtained the clinical follow-up data of 530 patients with ccRCC from TCGA-KIRC. According to the median expression of IRF6, these patients were divided into two groups, and results of log-rank test indicated that lower IRF6 expression was related to shorter Overall Survival (OS) and Disease Free Survival (DFS) (Figure 2C). Besides, the results of univariate Cox regression analysis demonstrated that older age, advanced tumor stage, distant metastasis, higher pathological stage and histological grade, and decreased IRF6 expression were associated with shorter OS in ccRCC patients. Moreover, the multivariate Cox regression analysis results indicated that older age, distant metastasis, higher pathological stage and histological grade, and decreased IRF6 expression (HR: 0.8524, 95%CI: 0.7614-0.9543, P=0.0056) were independently risk factors of shorter OS (Table 1). As far as DFS is concerned, advanced tumor stage, distant metastasis, higher pathological stage and histological grade, and decreased IRF6 expression were related to shorter DFS. The following multivariate Cox regression analysis results demonstrated that distant metastasis, higher pathological stage and decreased IRF6 expression (HR: 0.7024, 95%CI: 0.6087-0.8104, P<0.0001) were independently predictors of shorter DFS (Table 2).

### Verification of the expression of IRF6 in ccRCC and its prognostic value using clinical samples

**Figure 3:**
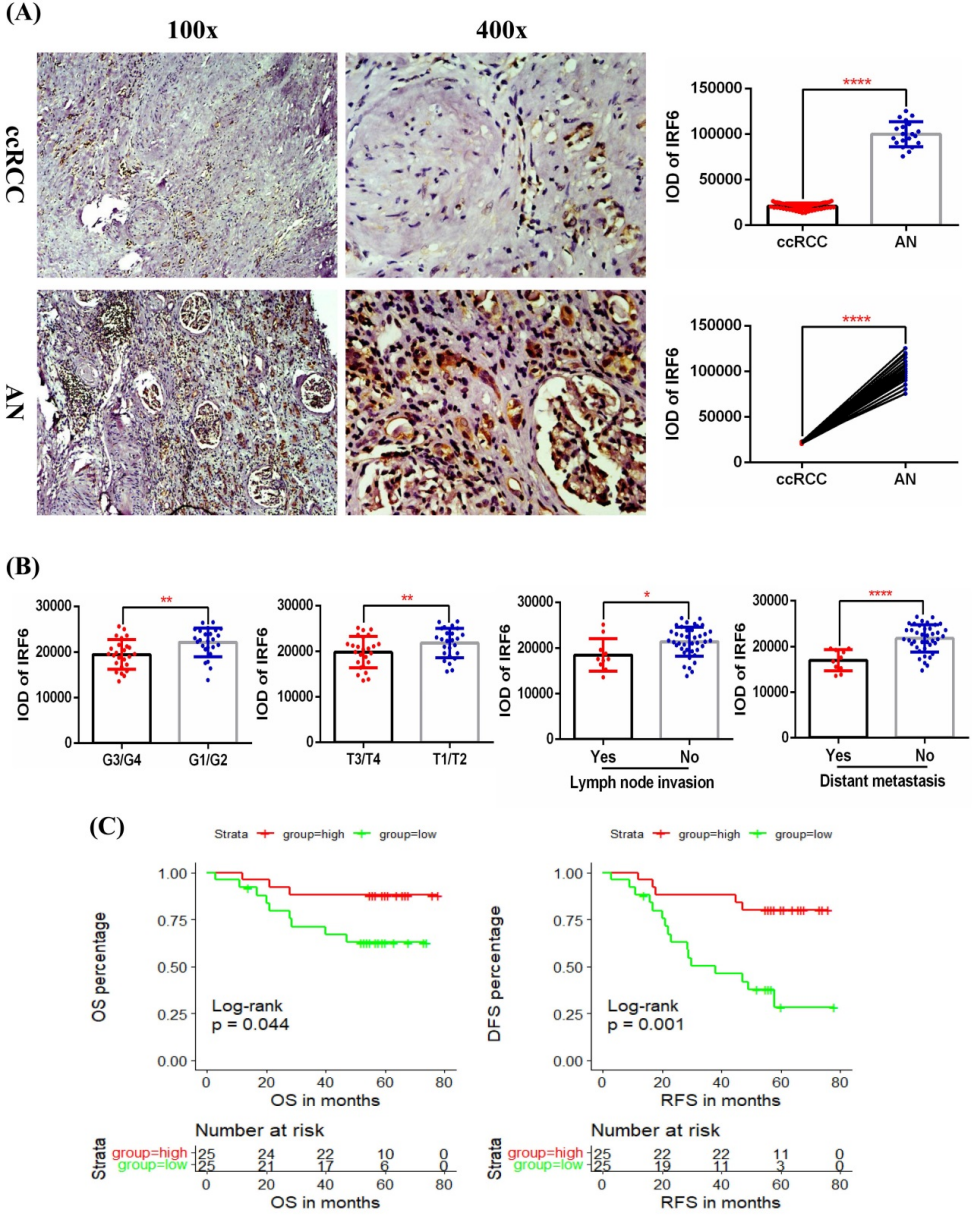
** Validation of the expression pattern and prognostic value of IRF6 in ccRCC. (A)** Comparison of IRF6 protein expression in 50 ccRCC tissues and 20 matched adjacent normal renal tissue using immunohistochemistry staining. **(B)** Decreased IRF6 expression was associated with higher histological grade, advanced tumor stage, lymph node invasion and distant metastasis. **(C)** IRF6 low expression group have shorter OS and DFS compared to IRF6 high expression group. Patients were separated into two groups according to the median cutoff of IRF6 expression. *p <0.05, **p <0.01, ****p < 0.0001.

In order to further clarify the expression level of IRF6 and its clinical role in ccRCC, we examined the protein expression of IRF6 in 50 ccRCC tissues and 20 matched adjacent normal renal tissues using immunohistochemistry staining, and the detailed clinicopathological characteristics of these 50 ccRCC patients was shown in Table 3. Our results confirmed that IRF6 expression was significantly decreased in ccRCC than that in adjacent normal renal tissues (Figure 3A), and decreased IRF6 expression was related to higher histological grade, advanced tumor stage, lymph node invasion, and distant metastasis (Figure 3B). Moreover, combined with the follow-up data of these 50 patients, we found that low IRF6 protein expression group had shorter OS and DFS than the relative IRF6 high expression group (Figure 3C). Taken together, the above results proved that IRF6 expression was downregulated in ccRCC, and lower IRF6 expression was associated with poorer prognosis.

### IRF6 expression was regulated by DNA methylation in ccRCC

**Figure 4:**
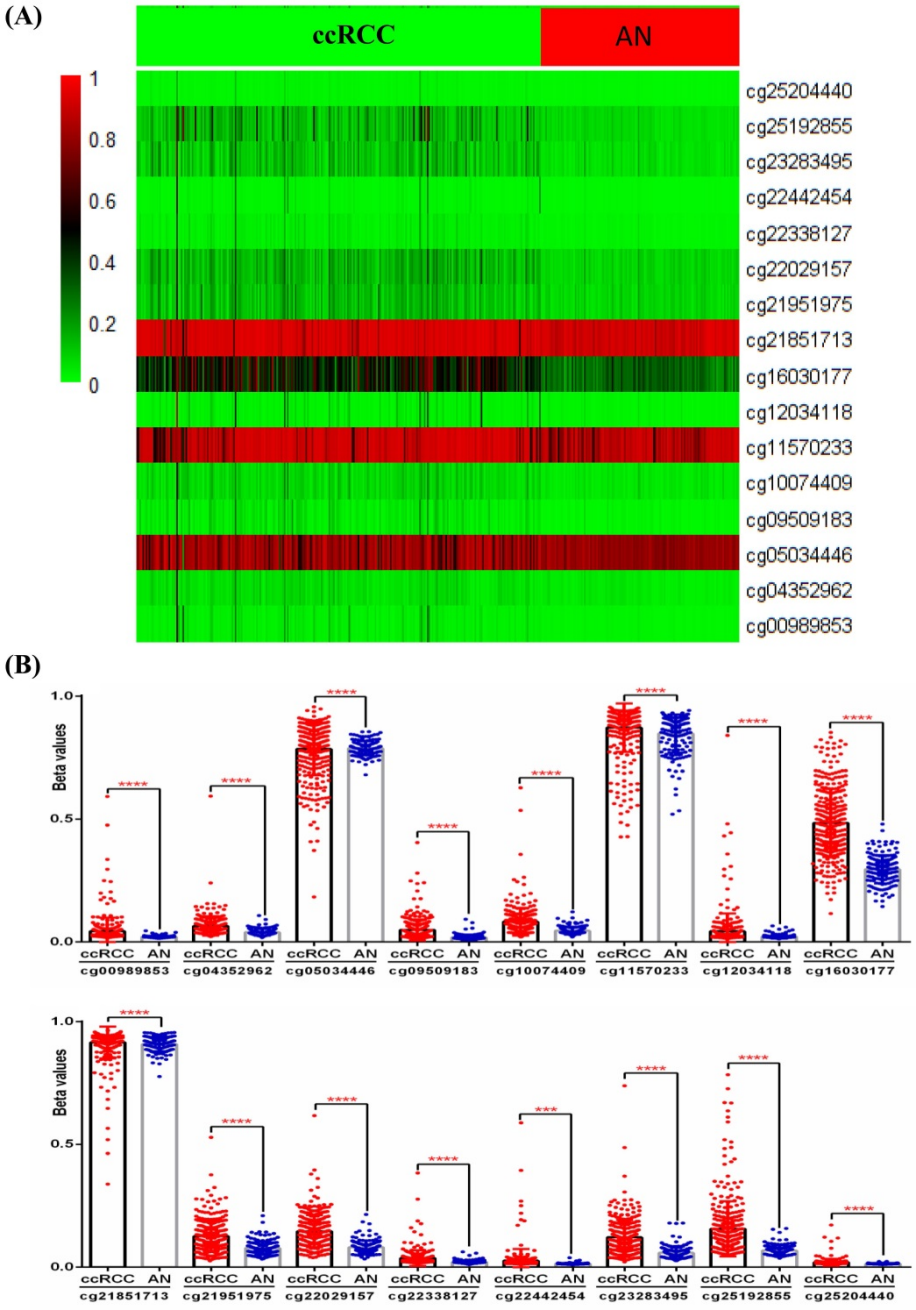
** Comparison of IRF6 DNA methylation status in ccRCC and adjacent normal renal tissues. (A)** Heatmap and **(B)** statistical comparison of the difference in methylation levels of 16 CpG sites of IRF6 DNA. ***p<0.001, ****p < 0.0001.

Although the above results determined the expression profile of IRF6 and its potential prognostic value in ccRCC, the mechanisms underlying its downregulation in ccRCC needs to be further explored. Thus, the DNA methylation data of 325 ccRCC tissues and 160 adjacent normal renal tissues from TCGA-KIRC was used to compare the methylation levels of 16 CpG sites (cg00989853, cg04352962, cg05034446, cg09509183, cg10074409, cg11570233, cg12034118, cg16030177, cg21851713, cg21951975, cg22029157, cg22338127, cg22442454, cg23283495, cg25192855 and cg25204440) of IRF6 DNA (Figure 4A), and the detailed information of these CpG sites was provided in Table 4. Our results indicated that the DNA methylation levels of all these 16 CpG sites were significantly increased in ccRCC than that in adjacent normal renal tissues (Figure 4B).

**Figure 5:**
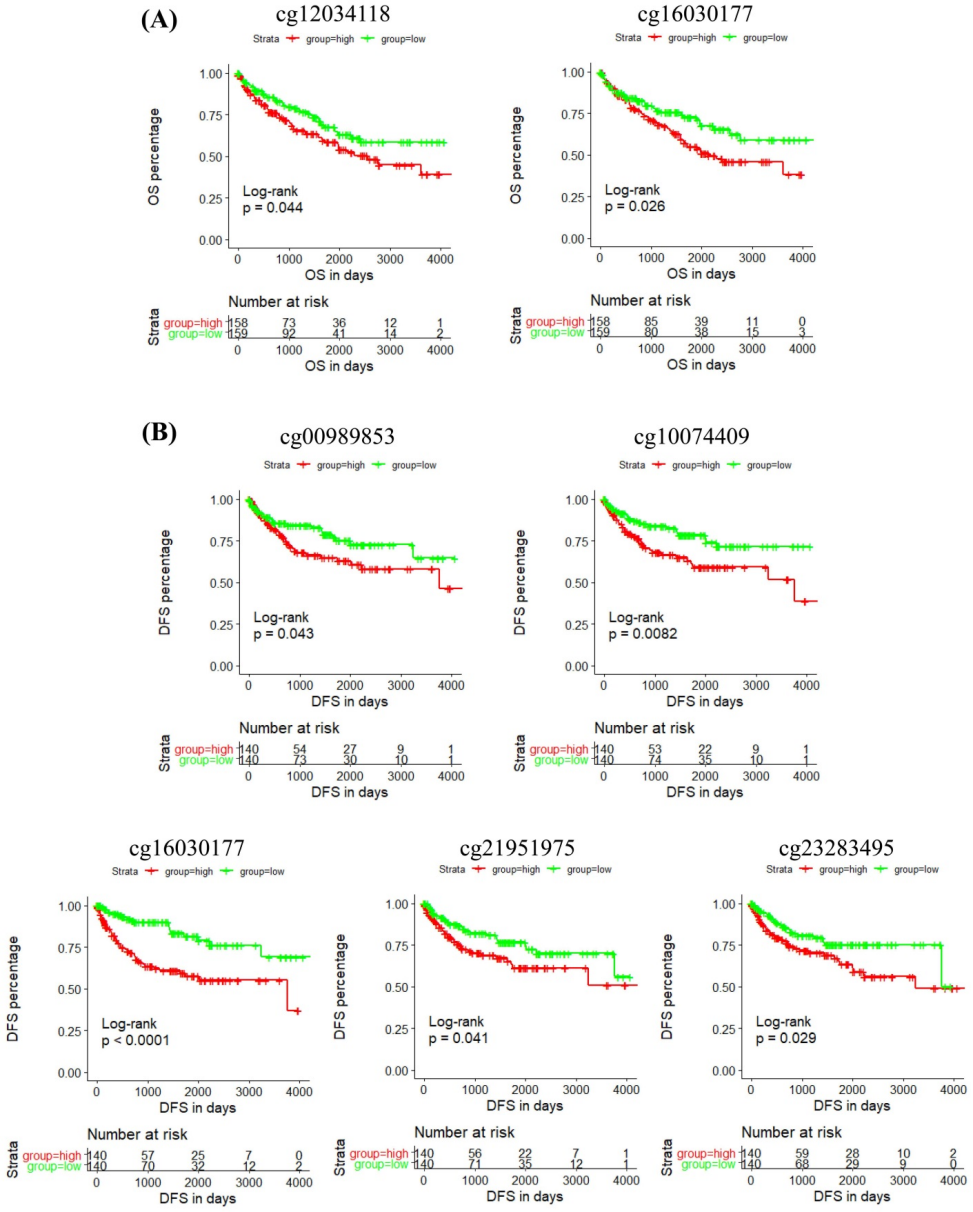
** The prognostic role of the CpG sites of IRF6 DNA in ccRCC. (A)** High methylation level groups of cg12034118 and cg16030177 had shorter OS than their related low methylation level groups. **(B)** High methylation level groups of cg00989853, cg10074409, cg16030177, cg21951975 and cg23283495 had shorter DFS than their related low methylation level groups. Patients were separated into two groups according to the median cutoff of the methylation levels of these CpG sites.

**Figure 6:**
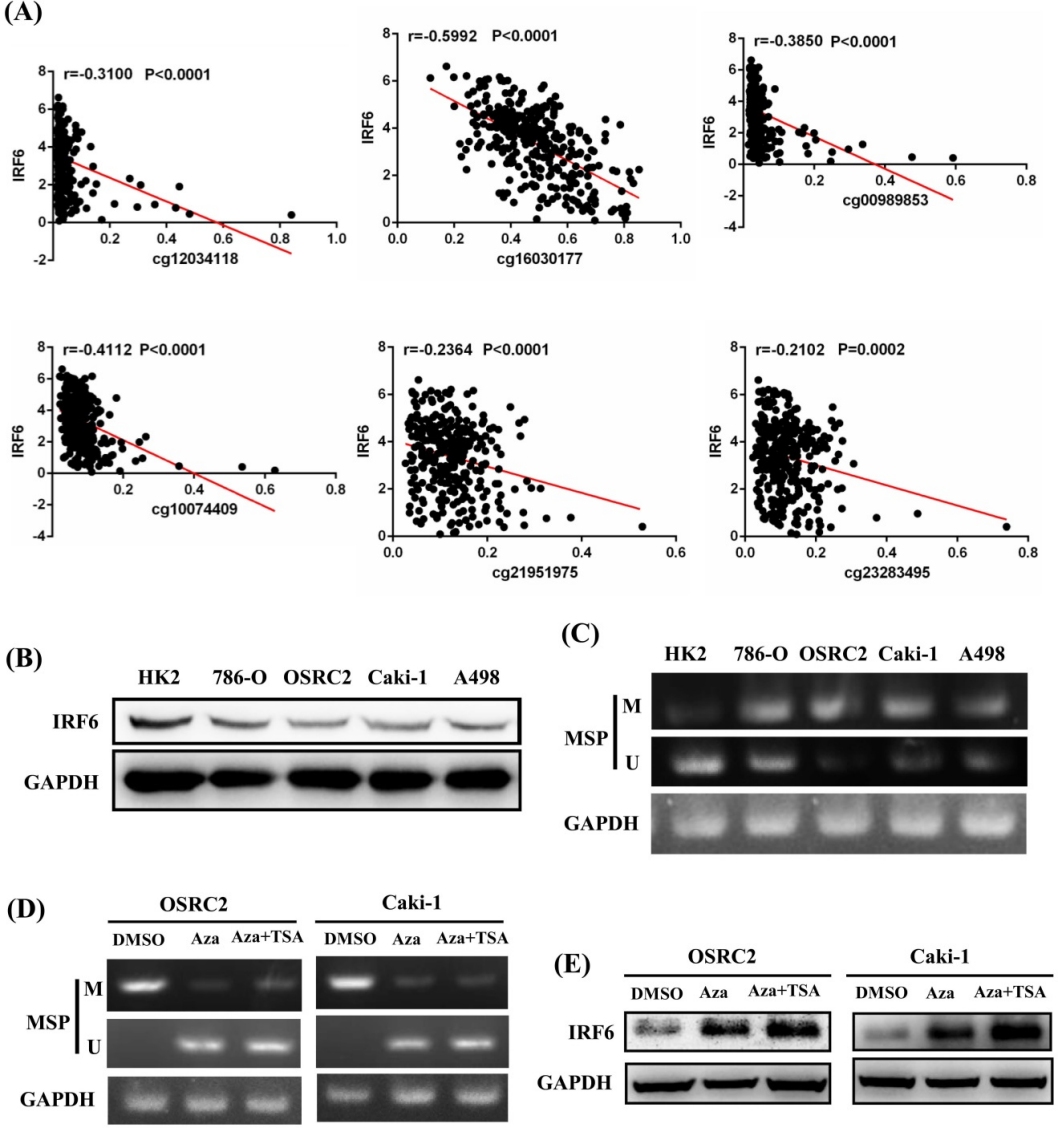
** IRF6 expression was regulated by DNA methylation in ccRCC. (A)** IRF6 expression was negatively correlated with the methylation levels of cg12034118, cg16030177, cg00989853, cg10074409, cg21951975 and cg23283495. **(B)** IRF6 expression was decreased in ccRCC cell lines. **(C)** IRF6 is hypermethylated in ccRCC cell lines compared to HK2 cell line. **(D)** DNA methylation level of IRF6 in OSRC2 and Caki-1 cells were significantly decreased after exposed to demethylating agents. **(E)** IRF6 expression in OSRC2 and Caki-1 cells was remarkably increased after exposed to demethylating agents. M = Methylated, U = Unmethylated.

In addition, the prognostic role of these CpG sites was also detected by Kaplan-Meier curve, and the results determined that high methylation level groups of cg12034118 and cg16030177 had significantly shorter OS than their related low methylation level groups (Figure 5A), and high methylation level groups of cg00989853, cg10074409, cg16030177, cg21951975 and cg23283495 had significantly shorter DFS than their related low methylation level groups (Figure 5B). Moreover, we analyzed the correlation between IRF6 expression and the methylation levels of these 6 CpG sites, and the linear correlation analysis results demonstrated that IRF6 expression was remarkably negatively correlated with the methylation levels of these 6 CpG sites (Figure 6A).

To verify the potential regulatory effect of DNA methylation on the expression of IRF6, MethPrimer 2.0 was used to detect the CpG islands of IRF6 DNA and design the corresponding MSP primers (Supplementary Figure 1). Our results showed that IRF6 protein expression was decreased in multiple ccRCC cell lines (786-O, OSRC2, Caki-1 and A498) compared to HK2 cell line (Figure 6B), and the DNA methylation levels of these ccRCC cell lines were increased than that in HK2 cell line (Figure 6C). Furthermore, the DNA methylation level of IRF6 in OSRC2 and Caki-1 cells were significantly decreased, and the expression of IRF6 in OSRC2 and Caki-1 cells was remarkably increased after exposed to demethylating agents (Figure 6D and 6E). To sum up, the above results confirmed that DNA hypermethylation played an important role in decreased IRF6 expression in ccRCC.

### IRF6 expression was associated with immune cells infiltration in ccRCC

**Figure 7:**
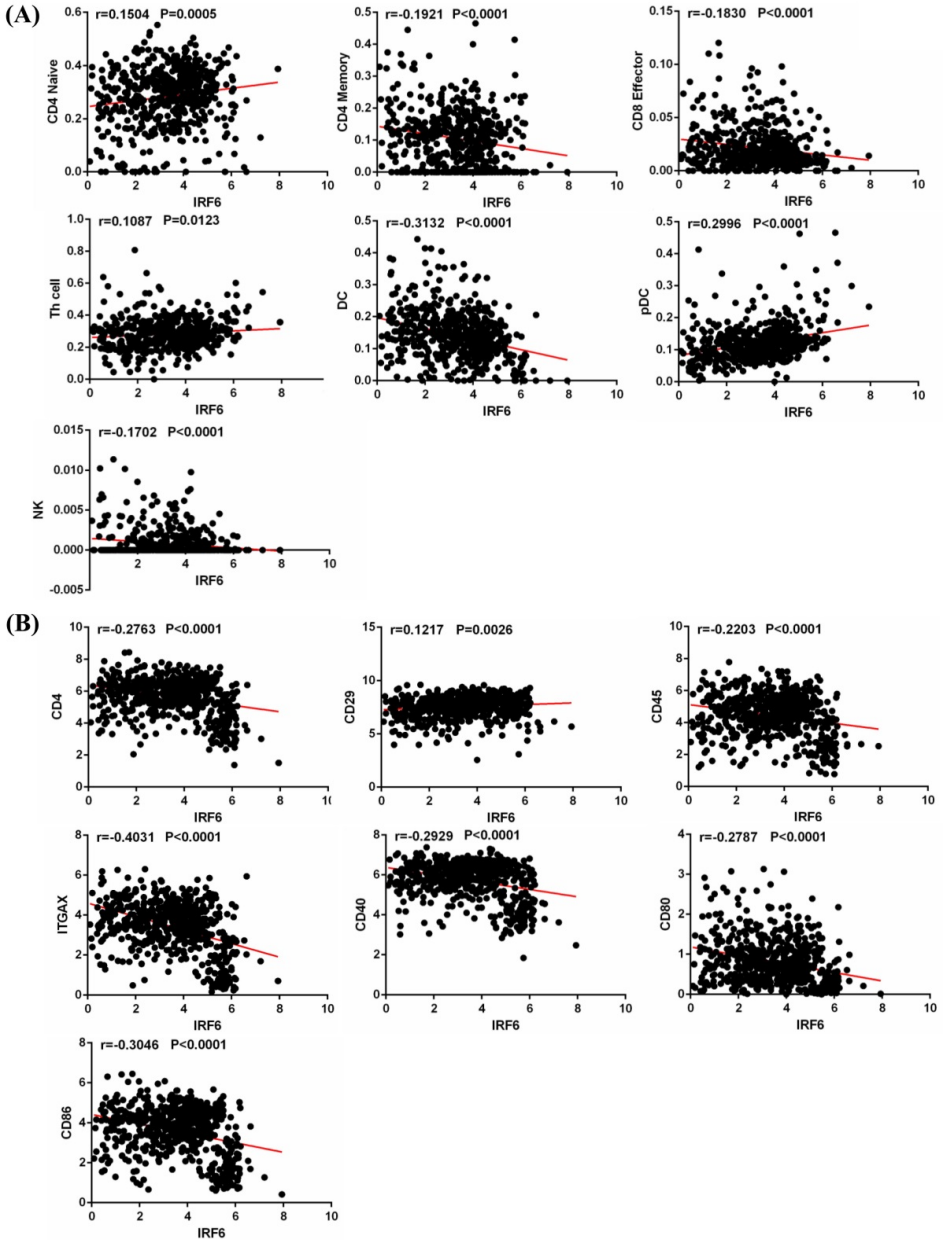
** IRF6 expression was associated with immune cells infiltration in ccRCC. (A)** IRF6 expression was significantly positively correlated with the infiltration levels of CD4 Naïve, Th cell and pDC, and negatively correlated with the infiltration levels of CD4 Memory, CD8 Effector, DC and NK cells. **(B)** IRF6 expression was negatively correlated with CD4, CD45, ITGAX, CD40, CD80 and CD86 expression, and positively correlated with CD29 expression.

Tumor infiltrating immune cells play an important role in regulating multiple important biological processes of tumor cells, and are related to the patient's prognosis and response to immunotherapy 17, 18. Thus, we obtained the infiltration data of 14 kinds of immune cells (B cells, CD4 Naïve, CD4 Memory, CD8 Naïve, CD8 Memory, CD8 Effector, Treg cell, Th cell, Monocytes CD16, Monocytes CD14, DC, pDC, NK and Plasma) from TIP, and analyzed their relationship with IRF6 expression. Results showed that IRF6 expression was significantly positively correlated with the infiltration level of CD4 Naïve, Th cell and pDC, and negatively correlated with the infiltration level of CD4 Memory, CD8 Effector, DC and NK cells (Figure 7A).

**Figure 8:**
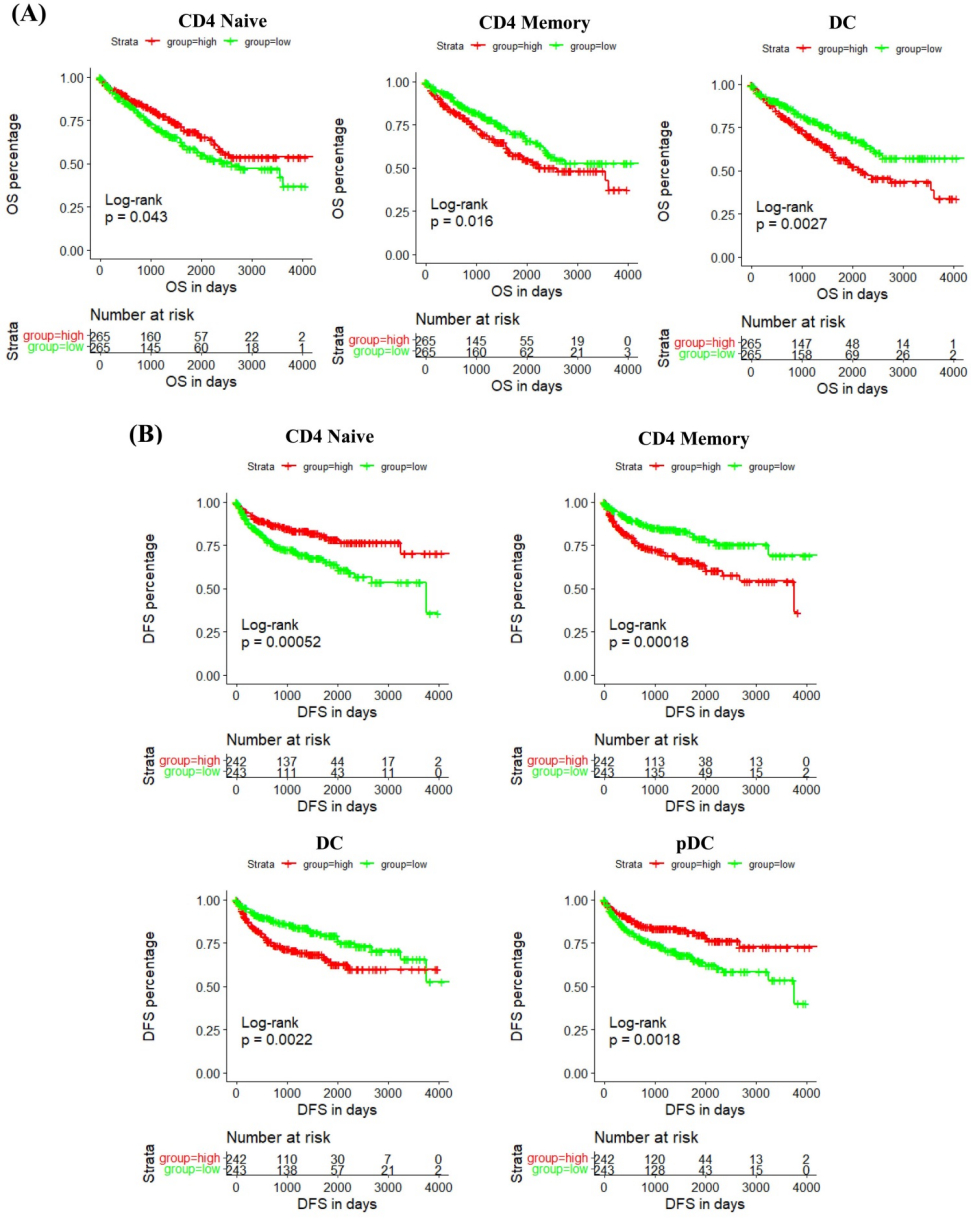
** The prognostic value of IRF6-related immune cells in ccRCC. (A)** Lower CD4 Naïve infiltration level and higher CD4 Memory and DC infiltration levels were related to shorter OS. **(B)** Lower CD4 Naïve and pDC infiltration levels and higher CD4 Memory and DC infiltration levels were related to shorter DFS.

In addition, we studied the correlation between IRF6 expression and these immune cells by considering the markers of immune cells. Results demonstrated that IRF6 expression was significantly correlated with the expression of markers of CD4 Naïve (CD4, CD45), CD4 Memory (CD4, CD45 and CD29), DC (ITGAX, CD40, CD80, CD86) and pDC (ITGAX) (Figure 7B). Moreover, the survival analysis results showed low CD4 Naïve infiltration and high CD4 Memory and DC infiltration groups had shorter OS (Figure 8A), and low CD4 Naïve and pDC infiltration and high CD4 Memory and DC infiltration groups had shorter DFS (Figure 8B).

**Figure 9:**
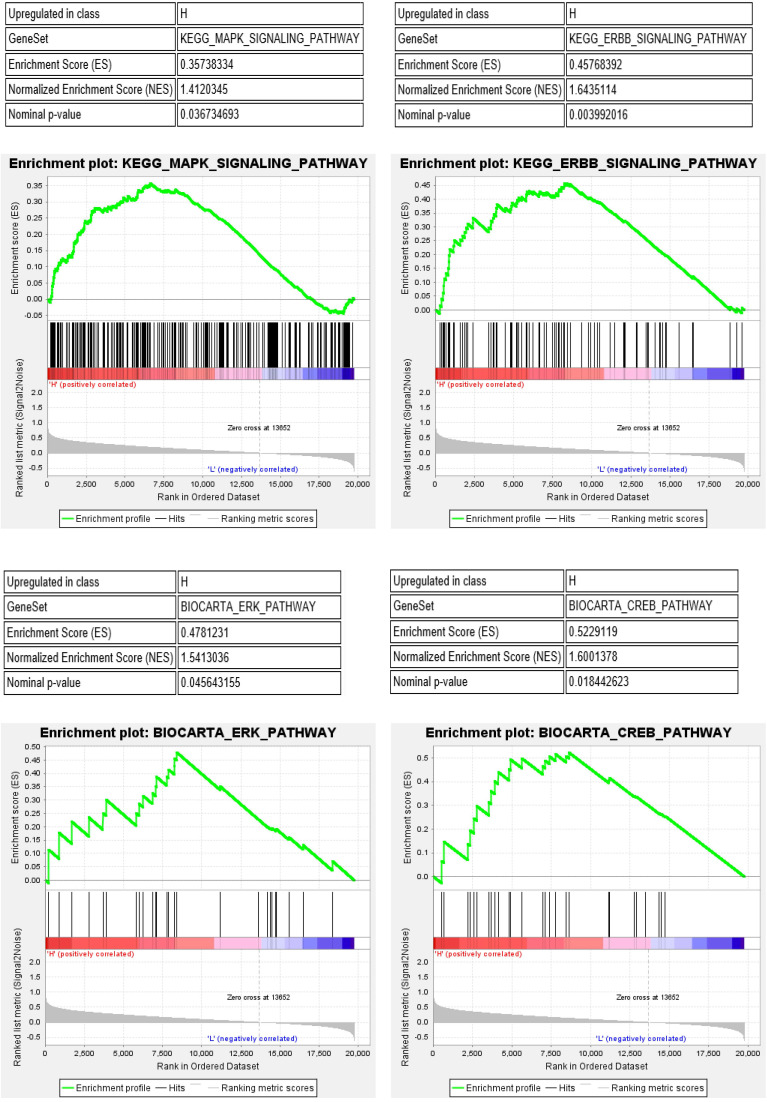
MAPK, ERBB, ERK and CREB pathways were significantly activated in the IRF6 high expression group.

Furthermore, we performed gene set enrichment analysis (GSEA) of the IRF6 high and low expression groups to study the potential molecular mechanism of IRF6. The results showed that MAPK, ERBB, ERK and CREB pathways, which play important roles in the regulation of immune cells in the tumor environment 19-22, were significantly activated in the IRF6 high expression group (Figure 9). Thus, the above results demonstrated that the decrease of IRF6 expression was related to the alterations of tumor immune cells infiltration, which might affect the prognosis of ccRCC patients.

## Discussion

Several recent studies have found that IRFs play an important function in kidney cancer and are closely related to the prognosis of patients. For instance, IRF8 functions as a tumor suppressor in RCC, and its mediated interferon signal pathway is involved in the pathogenesis of RCC 23; RCC patients with high IRF8 expression level have prolonged OS compared to patients with low level of IRF8 expression 24; IRF1 plays a pivotal role in the interferon-gamma-mediated-enhancement of Fas/CD95-mediated RCC cells apoptosis 25. Although previous studies have indicated that IRF6 also plays important roles in multiple tumors, including gastric cancer 13, nasopharyngeal carcinoma 14, squamous cell carcinoma 15 and cervical cancer 16, the expression pattern and prognostic value of IRF6 in ccRCC are still uncertain.

During our study, through joint analysis of the data from GEO and TCGA-KIRC databases, we found that IRF6 expression was significantly decreased in ccRCC than that in adjacent normal renal tissues, and decreased IRF6 expression was associated with advanced tumor stage, distant metastasis, higher histological grade and pathological stage, and worse prognosis. Besides, the multivariate Cox regression analysis results also indicated that decreased IRF6 expression was an independently risk factor predictor of shorter OS and DFS in ccRCC patients. Moreover, our immunohistochemical staining results verified the expression status of IRF6 and its prognostic value in 50 ccRCC tissues and 20 matched adjacent normal renal tissues.

Although the above results have determined the expression profile and prognostic value of IRF6 in ccRCC, the mechanisms underlying its downregulation remain unknown. It is reported that many tumor suppressor genes are partially or completely silenced due to DNA promoter hypermethylation and use of demethylating agents can restore the expression of many of these genes *in vitro* 26. Previous research have indicated that epigenetic IRFs inactivation plays a key role in the occurrence of gastric cancer, and the inhibition of DNA methylation can restore the anti-tumor activity of interferon by up-regulating IRF 27. Besides, study also showed that functional tumor suppressor IRF8 is frequently silenced by DNA methylation in multiple carcinomas 28, 29. Consistent with the above research results, our results also found that IRF6 expression was negatively correlated with the methylation levels of its CpG sites, and IRF6 expression was remarkably increased after exposed to demethylating agents in ccRCC cells.

Previous evidence suggested that tumor-associated immune cells in the tumor-associated microenvironment (TAM) play a crucial role in regulating multiple important biological processes of tumor cells. Various studies have proved that ccRCC is a highly immune infiltrating tumor, and tumor immune infiltration is closely related to the prognosis of RCC patients and the response to immunotherapy 17, 18, 30. Moreover, some studies have pointed out that IRFs were related to the regulation of immune cell development and immune responses in tumors 31. In the present study, our results demonstrated that IRF6 expression was significantly positively correlated with the infiltration level of CD4 Naïve, Th cell and pDC, and negatively correlated with the infiltration level of CD4 Memory, CD8 Effector, DC and NK cells. IRF6 expression was also significantly correlated with the expression of several markers of these immune cells, such as CD4, CD45, CD29, ITGAX, CD40, CD80 and CD86. Besides, decreased CD4 Naïve and pDC infiltration and increased CD4 Memory and DC infiltration were associated with poor prognosis in ccRCC. In addition, GSEA results showed that in the IRF6 high expression group, the MAPK, ERBB, ERK and CREB pathways, which play important roles in the regulation of immune cells in the TAM, were significantly activated. Therefore, the reduced expression of IRF6 may affect the prognosis of patients by regulating immune cells infiltration.

A recent study also pointed out that IRF6 expression was downregulated in renal carcinoma tissues and decreased IRF6 expression was associated with poor prognosis 32. However, these results were obtained by analyzing the public data of GEPIA and HPA databases, and were not verified with clinical renal carcinoma samples. Thus, our results are a further supplement and verification to the above viewpoints. Besides, the upstream regulatory mechanism of decreased IRF6 expression in ccRCC has not been reported. Previous study indicated that IRF6 expression was epigenetically repressed by DNA methylation in human bladder cancer cells UMUC3 33. In the current study, we also verified that the decrease of IRF6 expression was caused by DNA hypermethylation in ccRCC cells through MSP and demethylation analysis. Moreover, the relationship between IRF6 expression and tumor immune cells infiltration has not been elucidated. Although the present study was based on TCGA database to explore the relationship between the expression level of IRF6 and the infiltration levels of multiple immune cells in ccRCC, the analysis results have a suggestive effect on our further study of the downstream regulatory mechanism of IRF6 in the near future.

In addition, our study still has some limitations. Although our results determined that IRF6 expression was significantly correlated immune cells infiltration, the specific regulation mechanism of IRF6 on immune cells infiltration and immune response is vague. Therefore, in the near future, more experiments such as immunofluorescence and flow cytometry are needed to screen a kind of specific immune cell to further determine the correlation of IRF6 expression with immune cells infiltration in ccRCC. Nevertheless, our study has determined the expression profile and clinical roles of IRF6 in ccRCC through various databases and laboratory experiments, and these findings may provide a theoretical basis for the development of new therapeutic target.

## Conclusions

In conclusion, our study confirmed that IRF6 expression was significantly reduced in ccRCC and DNA hypermethylation played an important role in decreased IRF6 expression. In addition, the decrease of IRF6 was related to the poor prognosis of ccRCC patients and the alterations of tumor immune cells infiltration.

## Supplementary Material

Supplementary figure.

## Figures and Tables

**Table 1:** Univariate and multivariate Cox regression analysis of OS in TCGA ccRCC patients

Parameters	Univariate analysis				Multivariate analysis			
	P	HR	95%CI		P	HR	95%CI	
			Lower	Upper			Lower	Upper
**Age**								
<70 (n=383)		1.0000				1.0000		
≥70 (n=128)	**<0.0001**	1.9550	1.4300	2.6740	**<0.0001**	2.1527	1.5601	2.9705
**Gender**								
Female (n=176)		1.0000						
Male (n=335)	0.7970	0.9595	0.7001	1.3150				
**Tumor stage**								
T1/T2 (n=325)		1.0000				1.0000		
T3/T4 (n=186)	**<0.0001**	3.0560	2.2460	4.1570	0.5236	0.8167	0.4383	1.5216
**Distant metastasis**								
No (n=432)		1.0000				1.0000		
Yes (n=79)	**<0.0001**	4.3520	3.1860	5.9440	**<0.0001**	2.3672	1.5974	3.5081
**Pathological stage**								
I/II (n=307)		1.0000				1.0000		
III/IV (n=204)	**<0.0001**	3.6810	2.6710	5.0740	**0.0147**	2.4443	1.1920	5.0126
**Histological grade**								
G1/G2 (n=233)		1.0000				1.0000		
G3/G4 (n=278)	**<0.0001**	2.6740	1.8910	3.7820	**0.0112**	1.6101	1.1142	2.3266
**IRF6** (continuous, n=511)	**<0.0001**	0.7135	0.6415	0.7936	**0.0056**	0.8524	0.7614	0.9543

**Table 2:** Univariate and multivariate Cox regression analysis of DFS in TCGA ccRCC patients

Parameters	Univariate analysis				Multivariate analysis			
	P	HR	95%CI		P	HR	95%CI	
			Lower	Upper			Lower	Upper
**Age**								
<70 (n=349)		1.0000						
≥70 (n=118)	0.7770	1.0650	0.6895	1.6450				
**Gender**								
Female (n=162)		1.0000						
Male (n=305)	0.0680	1.4780	0.9715	2.2480				
**Tumor stage**								
T1/T2 (n=309)		1.0000				1.0000		
T3/T4 (n=158)	**<0.0001**	4.5180	3.0690	6.6510	0.9863	1.0058	0.5195	1.9474
**Distant metastasis**								
No (n=414)		1.0000				1.0000		
Yes (n=53)	**<0.0001**	12.0300	8.0760	17.9100	**<0.0001**	4.8695	3.0164	7.8609
**Pathological stage**								
I/II (n=295)		1.0000				1.0000		
III/IV (n=172)	**<0.0001**	6.8300	4.4650	10.4500	**0.0042**	3.2464	1.4495	7.2708
**Histological grade**								
G1/G2 (n=219)		1.0000				1.0000		
G3/G4 (n=248)	**<0.0001**	3.3350	2.1480	5.1760	0.1074	1.4701	0.9197	2.3500
**IRF6** (continuous, n=467)	**<0.0001**	0.6350	0.5565	0.7246	**<0.0001**	0.7024	0.6087	0.8104

**Table 3:** The clinical and pathological characteristics of 50 ccRCC patients that used for validation

Clinicopathologic characteristics	N (%)
**Gender**	
Male	36 (72.0)
Female	14 (28.0)
**Age**	
≤60	28 (56.0)
>60	22 (44.0)
**BMI**	
<18.5	2 (4.0)
≥18.5, <24	19 (38.0)
≥24	29 (58.0)
**Tumor size**	
<5 cm	11 (22.0)
≥5 cm, <10 cm	30 (60.0)
≥10cm	9 (18.0)
**Tumor stage**	
T1/T2	25 (50.0)
T3/T4	25 (50.0)
**Histological grade**	
Grade 1/2	25 (50.0)
Grade 3/4	25 (50.0)
**Metastasis**	
No	40 (80.0)
Yes	10 (20.0)
**Lymph node invasion**	
No	40 (80.0)
Yes	10 (20.0)
**Overall survival**	
Alive	38 (76.0)
Dead	12 (24.0)
**Replase free survival**	
Non-replased	29 (58.0)
Replased	21 (42.0)

**Table 4:** The detailed information of 16 CpG sites in IRF6 DNA

Composite Element REF	Chromosome	Start	End	Feature_Type
cg00989853	chr1	209806000	209806001	Island
cg04352962	chr1	209806411	209806412	S_Shore
cg05034446	chr1	209788527	209788528	S_Shelf
cg09509183	chr1	209806279	209806280	Island
cg10074409	chr1	209806032	209806033	Island
cg11570233	chr1	209805052	209805053	N_Shore
cg12034118	chr1	209806142	209806143	Island
cg16030177	chr1	209805766	209805767	N_Shore
cg21851713	chr1	209802937	209802938	N_Shelf
cg21951975	chr1	209806388	209806389	S_Shore
cg22029157	chr1	209806320	209806321	Island
cg22338127	chr1	209806227	209806228	Island
cg22442454	chr1	209806125	209806126	Island
cg23283495	chr1	209806434	209806435	S_Shore
cg25192855	chr1	209805938	209805939	N_Shore
cg25204440	chr1	209806253	209806254	Island

## Abbreviations

ccRCC: clear cell renal cell carcinoma
IRFs: Interferon regulatory factors
SCC: squamous cell carcinoma
OS: overall survival
DFS: Disease Free Survival
GEO: Gene Expression Omnibus
TCGA-KIRC: The Cancer Genome Atlas-Kidney Renal Clear Cell Carcinoma
TIP: Tracking Tumor Immunophenotype
MSP: Methylation-specific PCR
TAM: tumor associated microenvironment
GSEA: gene set enrichment analysis
