# Supplementary material for: Decreased interferon regulatory factor 6 expression due to DNA hypermethylation predicts an unfavorable prognosis in clear cell renal cell carcinoma
Source: J Cancer. 2021 Sep 13;12(22):6640–55. doi: 10.7150/jca.62394 (PMC8518015; doi:10.7150/jca.62394)

Supplementary Material

Supplementary Figure 1. The prediction of CpG islands in IRF6 DNA. The horizontal axis of the curved lines represents the input sequence of IRF6 DNA, and the vertical axis of the curved lines represents GC percentage. TSS: Transcription Start Sites.

Supplementary Figure 1

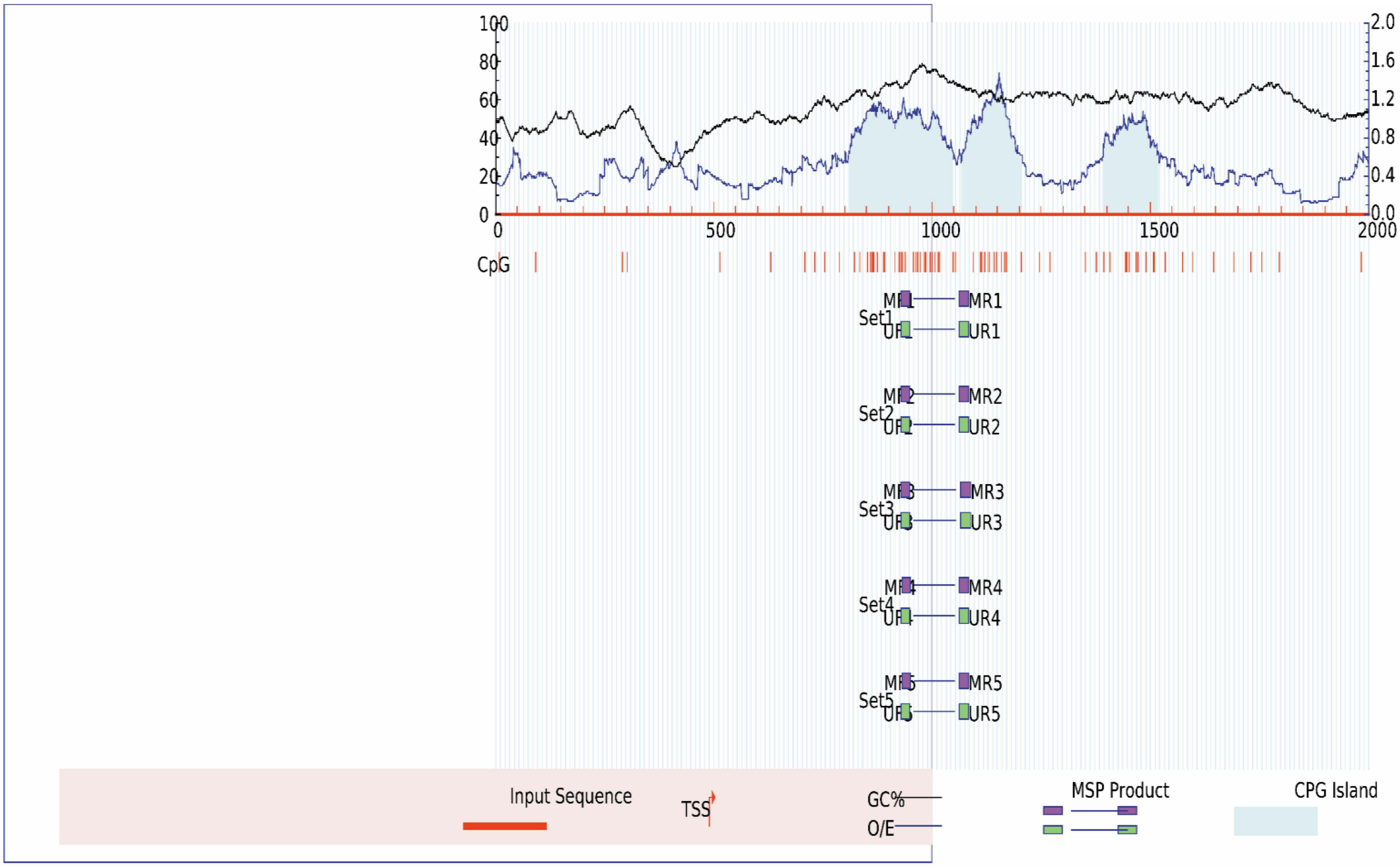

Supplement: Supplementary file 1 — Supplementary figure. [file jcav12p6640s1.pdf]
